# Is Life Extension Today a Faustian Bargain?

**DOI:** 10.3389/fmed.2017.00215

**Published:** 2017-11-29

**Authors:** S. Jay Olshansky

**Affiliations:** ^1^University of Illinois at Chicago, Chicago, IL, United States

**Keywords:** aging, mortality, longevity, health, biology

The legend of Faust is a thought-provoking tale from the middle ages, which has a surprising connection to the world of aging today. The story is both inspiring and tragic at the same time. In one famous version of the tale from Goethe, Faust is an idealist scholar that becomes disillusioned with his limits to knowledge. Bored and suicidal, Faust becomes the target of the devil Mephistopheles who says he can satisfy Faust’s desire for unlimited knowledge and also promises him youth, pleasures of the flesh, and magical powers—for a predetermined period. In exchange, after the allotted time, the devil will claim Faust’s soul and forever be enslaved. The story of Faust has become a metaphor for a promise or tradeoff that at first seems appealing, but with time is revealed to be a bad bargain.

The story of human aging and the modern rise in longevity has remarkable correlates to the story of Faust, but with some interesting twists. Here’s the connection. The first longevity revolution that began in the middle of the nineteenth century occurred primarily because of gains made against infant and child mortality resulting from advances in basic public health. This was followed by reductions in death rates from cardiovascular disease late in the twentieth century. A quantum leap in life expectancy of 30 years ensued at lightning speed. Humanity displayed a collective sigh of relief as infectious diseases waned—our children had finally been saved. Nothing in history has ever come close to the magnitude of this benefit to humanity. While there is no disputing the value of life and health extension from the first longevity revolution, rarely does something so desirable come without a Faustian-like price.

Along with 30 years of additional life and the opportunity to see almost all our children live long enough to have families of their own, humanity also witnessed a subsequent dramatic escalation in the prevalence of age-related chronic, fatal and disabling diseases and their attendant costs and heartache. That is, we now live long enough to experience the aging of our bodies. If Mephistopheles had been by our side in 1850 to explain what humanity would receive in exchange for longer lives, he would have simply said look around—the tradeoff is visible now among the handful of people fortunate enough to escape the usual scourges of childhood. The price humanity had to pay for 30 years of additional life was the rise in heart disease, cancer, stroke, Alzheimer’s disease, Parkinson’s, diabetes, and a long list of non-fatal disabling conditions. In retrospect, it was worth every part of the bargain.

But Mephistopheles isn’t done with us. Like a street magician that lets you win the first game, and then sucks you into a bigger con with larger stakes, or a drug dealer that gets you hooked with free samples, the next much costlier offer is before us now. We’ve had our taste of longevity, but now we want more—much more at any cost, and Mephistopheles knows this.

With life itself as the most precious commodity there is, it’s easy to see the next con. The first is the rise of what has become known as the antiaging industry—a multibillion dollar enterprise designed to convince us that the secret to the fountain of youth is already within our grasp (1, 2). Pay dearly for their elixirs now and wait for the promise of a longer life to appear decades later. What do you think the chances are that your investment will pay off? The catch is that the alleged benefits don’t appear, if at all, until after the longevity salesmen has left the scene and pocketed your cash. This longevity racket has been around from the beginning of time, and I refer to this industry as the second oldest profession. What’s different today from the alchemists of the middle ages, or the gland grafters of the early twentieth century, or the hormone vendors now peddling their wares, is the rise of the scientific study of aging and genuine opportunity offered for healthy life extension. The modern practitioners of anti-aging medicine try and sell the public what appear to be genuine scientific interventions based on real science, before they’re proven to be safe and efficacious. This idea is best personified in an early twentieth century quote from Alan Valentine: “*whenever science makes a discovery, the devil grabs it while the angels are debating the best way to use it*.”

The second response to an insatiable desire for more life is also predictable, but the danger could be an even worse Faustian bargain than that posed by the antiaging industry. The method used to manufacture the first longevity revolution is known as the “infectious disease model”—that is, as soon as a disease appears, attack it with everything in the medical arsenal. Beat the disease down, and once you succeed, push the patient out the door until they face their next challenge; then beat that one down. The formula is simple—repeat until failure. This model was perfect for infectious diseases and effective at first for chronic degenerative diseases, and no doubt there is still progress to be made, but evidence has emerged that this approach is likely to run out of steam (3–7). The application of an infectious disease model to chronic fatal and disabling diseases associated with aging is Mephistopheles latest “bargain.” The irony behind this new bargain (otherwise known as the current medical model of disease) is that the medical community advocating for disease eradication doesn’t even recognize the health consequences of success.

The bargain today is crystal clear—we’re being offered incrementally smaller amounts of survival time at a very high cost, and the prospect that most of the additional months and years of life will be riddled with frailty and disability. Keep in mind that the human body has no designer; it was not constructed for long-term use; and our Achilles heels are already visible—neurological conditions such as Alzheimer’s disease and related conditions are associated with non-replicating neurons; and muscles and joints have a difficult time navigating the ravages of biological time. *The Faustian bargain before us now is, in exchange for small doses of additional life, humanity will experience a suite of fatal and disabling conditions expressed at later ages that rob us of what we hold most precious – our mental and physical functioning*.

What’s the solution? Don’t sign the contract! A clue about what we should do instead was presented to us decades ago. In the mid-1950s, gerontologist McKay et al. suggested that attacking aging itself rather than the diseases associated with it offered the greatest hope in warding off the infirmities of old age (8). Some 20 years later, Bernie Strehler coined the term “gerontogeny” to convey the same message (9). The first formal discussion of delayed aging among scientists appeared in Extending the Human Life Span: Social Policy and Social Ethics, published by Neugarten and Havighurst (10). That book was a product of a three-year project on the future of aging funded by the National Science Foundation, culminating in a conference in 1976.

Conference participants were asked to discuss several questions: should the science of biogerontology be devoted to improving older people’s quality of life? Or should it extend the lifespan of the human species? If lifespan is extended, what would be its deleterious and beneficial effects on society? How much money should be allocated to research addressed directly to extending the human lifespan? What social and ethical implications would follow from a “magic elixir” that would extend active life expectancy by 15 to 20 years?

At the conference, it was noted that the longevity revolution in the twentieth century brought decades of healthy life, and contributed substantially to our nation’s economic growth. But all was not rosy. Conference participants were acutely aware that extended lives came at a price—rapid increases in chronic fatal and disabling diseases. Some scientists there argued we should not pursue life extension as a national goal because the result would be an increase in the number and proportion of people requiring acute nursing care. Gerontological Society president George Sacher expressed concern that extending life without extending health would result in a disproportionate number of years of disease and disability for the 10% of the population living the longest. But most conference participants agreed with James Goddard (former Commissioner of the US Food and Drug Administration), who argued that healthy life extension should be a national goal requiring political support and strong vested interests. Although the National Institute on Aging (NIA) was formed just before the Neugarten conference, the focus of modern medicine (and most the NIA budget since its beginning) has been centered on the disease model rather than the delayed-aging model. Advice from Neugarten conference participants to escalate the attack on aging, as well as to battle against major diseases, was not followed.

In 2006, my colleagues and I extended this line of reasoning by coining the phrase “the Longevity Dividend” to describe the economic and health benefits that would accrue to individuals and societies if we extend healthy life by slowing the biological processes of aging (11–13). This idea was distinctive because we proposed to extend healthy life by shifting our emphasis from disease management to delayed aging. Four factors led to this proposal: rapid increases in life expectancy since the late 1970s; accelerated population aging; and rapid increases in chronic fatal and disabling diseases. These three occurred rapidly in developed nations, and developing nations are catching up. The fourth factor was the most important—recent advances in biogerontology suggested that it is plausible to delay aging in people. (For a summary of this line of reasoning, see asaging.org/blog/delay_aging_further_reading.) A recent article in *Nature* suggests that “senolytics” may offer a unique opportunity to forestall the ravages of aging through the systematic elimination of cells that are still alive, but which no longer function normally (14).

The Longevity Dividend is an approach to public health based on a broader strategy of fostering health for all generations by developing a new horizontal model to health promotion and disease prevention. Unlike the current vertical approach to disease that targets individual disorders as they arise, the Longevity Dividend model seeks to prevent or delay the root causes of disease and disability by attacking the one main risk factor for them all—biological aging. Evidence in models ranging from invertebrates to mammals suggests that all living things have biochemical mechanisms influencing how quickly they age, and these mechanisms are adjustable.

Slowing down the processes of aging—even by a moderate amount—will yield dramatic improvements in health for current and future generations (12). Advances in the scientific knowledge of aging may thus create new opportunities that allow us, and generations to follow, to live healthier and longer lives than our predecessors. Bernice Neugarten and her colleagues had their finger on the right pulse decades ago—it just took 35 years for the scientific study of aging to catch up. By embracing a new model for health promotion and disease prevention in the twenty-first century, we can give the gift of extended health and economic wellbeing to current and all future generations. What is the cost of this new more effective model of primary prevention that will save the world trillions in health care costs? A fraction of the basic research cost required to create sixth generation fighter jets; or the salary from just one quarterback in the National Football League.

The case can now be made that delayed aging could be the most efficient method of achieving primary prevention available to us in this century. A large-scale, concerted, and coordinated effort is now underway to translate the science behind the Longevity Dividend Initiative, also known as Geroscience, into real-world clinical trials and a suite of therapeutic interventions (15–18).

So, are we giving up too much to satisfy our insatiable appetite for more life? Is today’s Faustian bargain worth it?

## Author Contributions

The author confirms being the sole contributor of this work and approved it for publication.

## Conflict of Interest Statement

The author declares that the research was conducted in the absence of any commercial or financial relationships that could be construed as a potential conflict of interest.
